# Supplementary figures and images for: Characterization of nanomaterials synthesized from Spirulina platensis extract and their potential antifungal activity
Source: PLoS One. 2022 Sep 16;17(9):e0274753. doi: 10.1371/journal.pone.0274753 (PMC9481030; doi:10.1371/journal.pone.0274753)

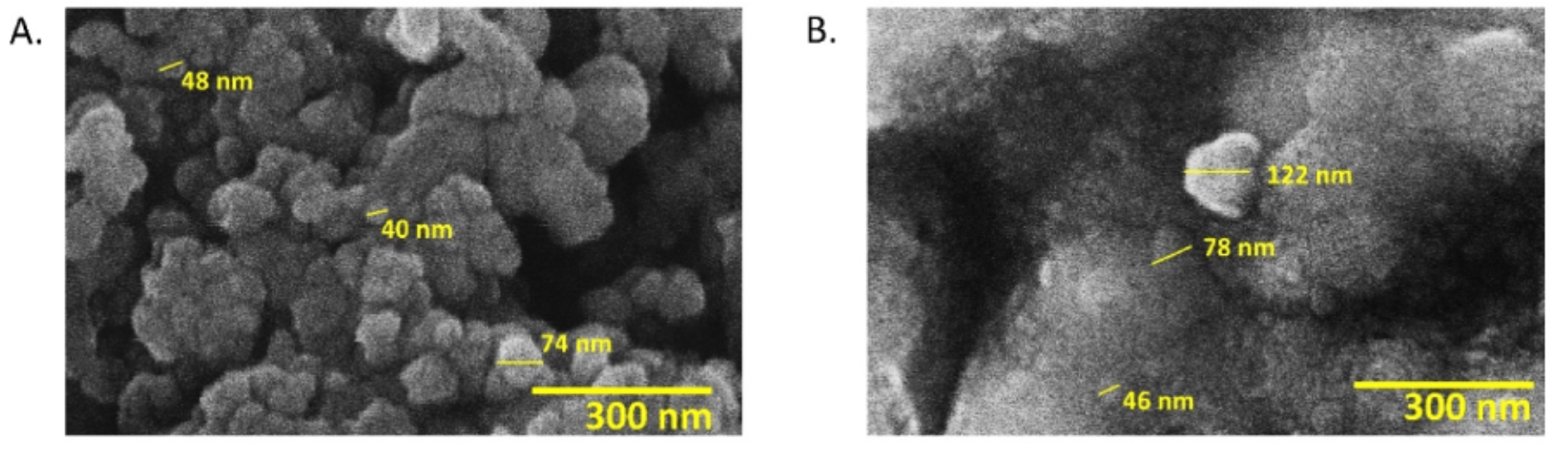

Supplement: S1 Fig — SEM images of Ag NPs (A) before calcination, (B) after calcination. (TIFF) [file pone.0274753.s001.tiff]

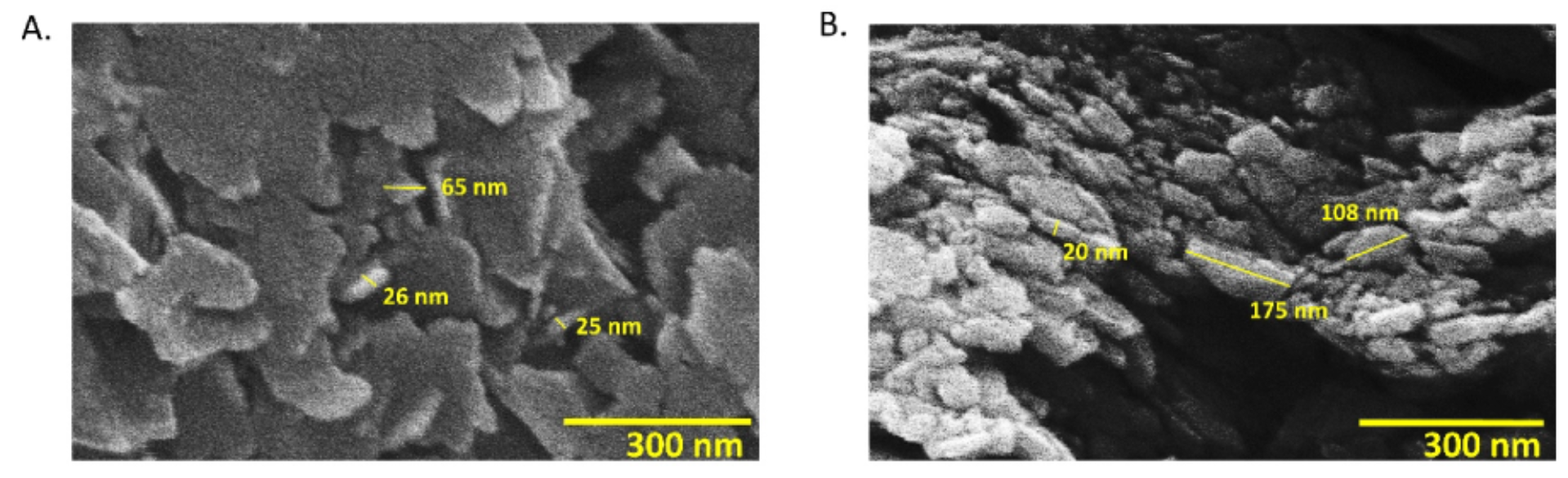

Supplement: S2 Fig — SEM images of TiO2 NPs (A) before calcination, (B) after calcination. (TIFF) [file pone.0274753.s002.tiff]

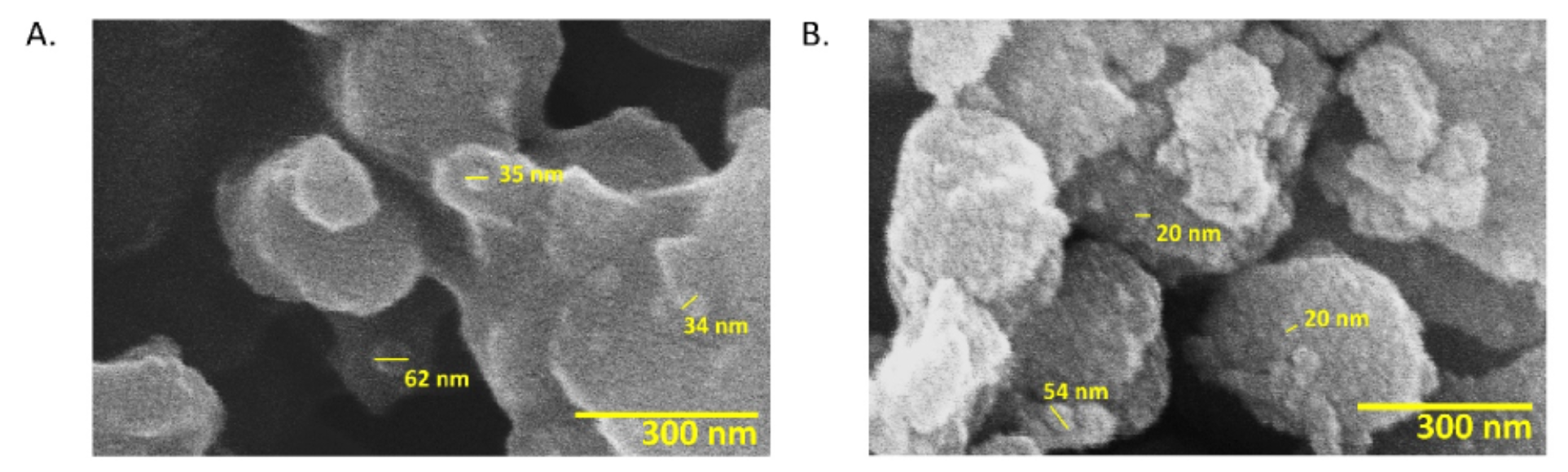

Supplement: S3 Fig — SEM images (A) Co(OH)2 (before calcination), (B) Co3O4 (after calcination). (TIFF) [file pone.0274753.s003.tiff]

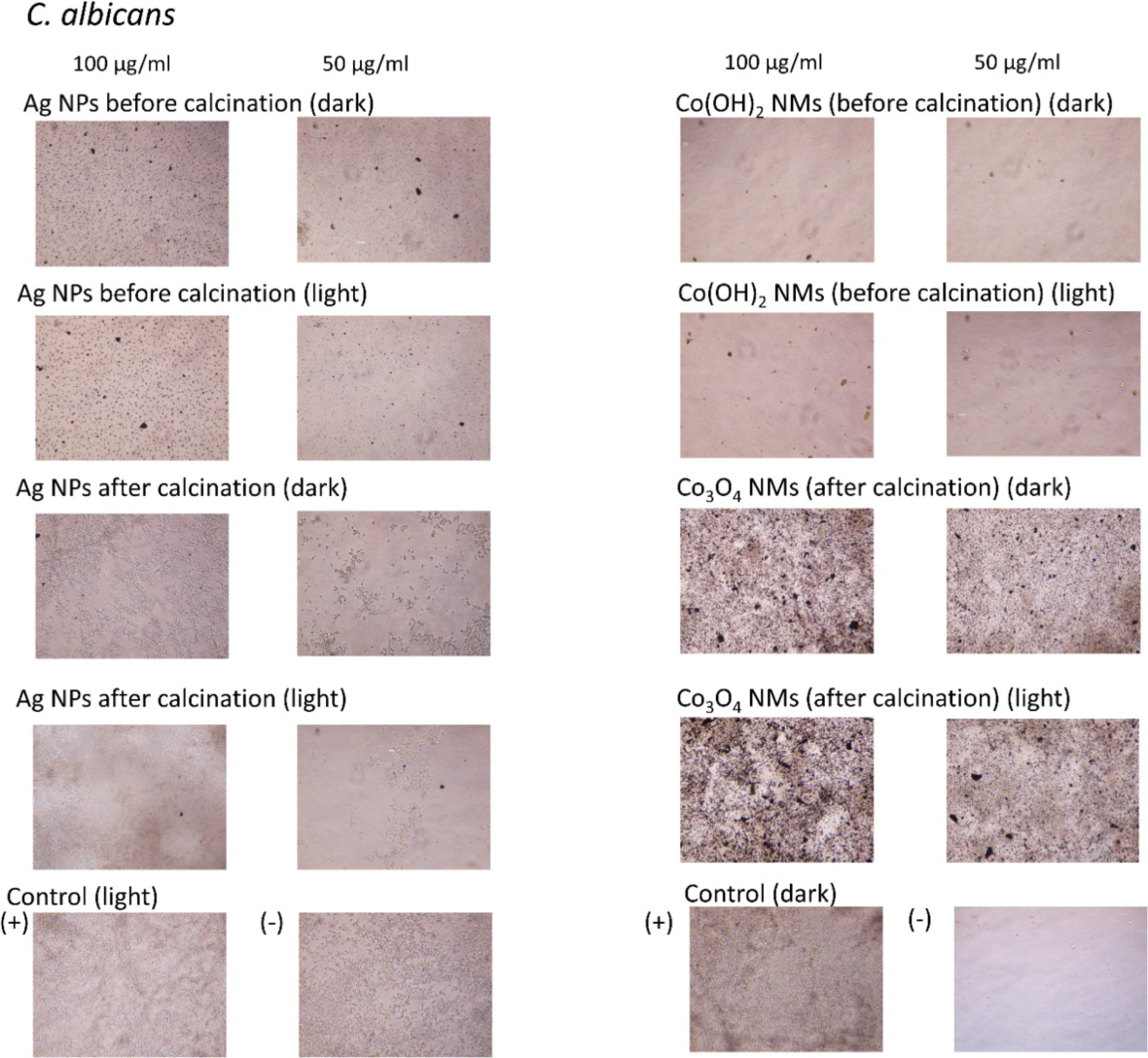

Supplement: S4 Fig — (TIFF) [file pone.0274753.s004.tiff]

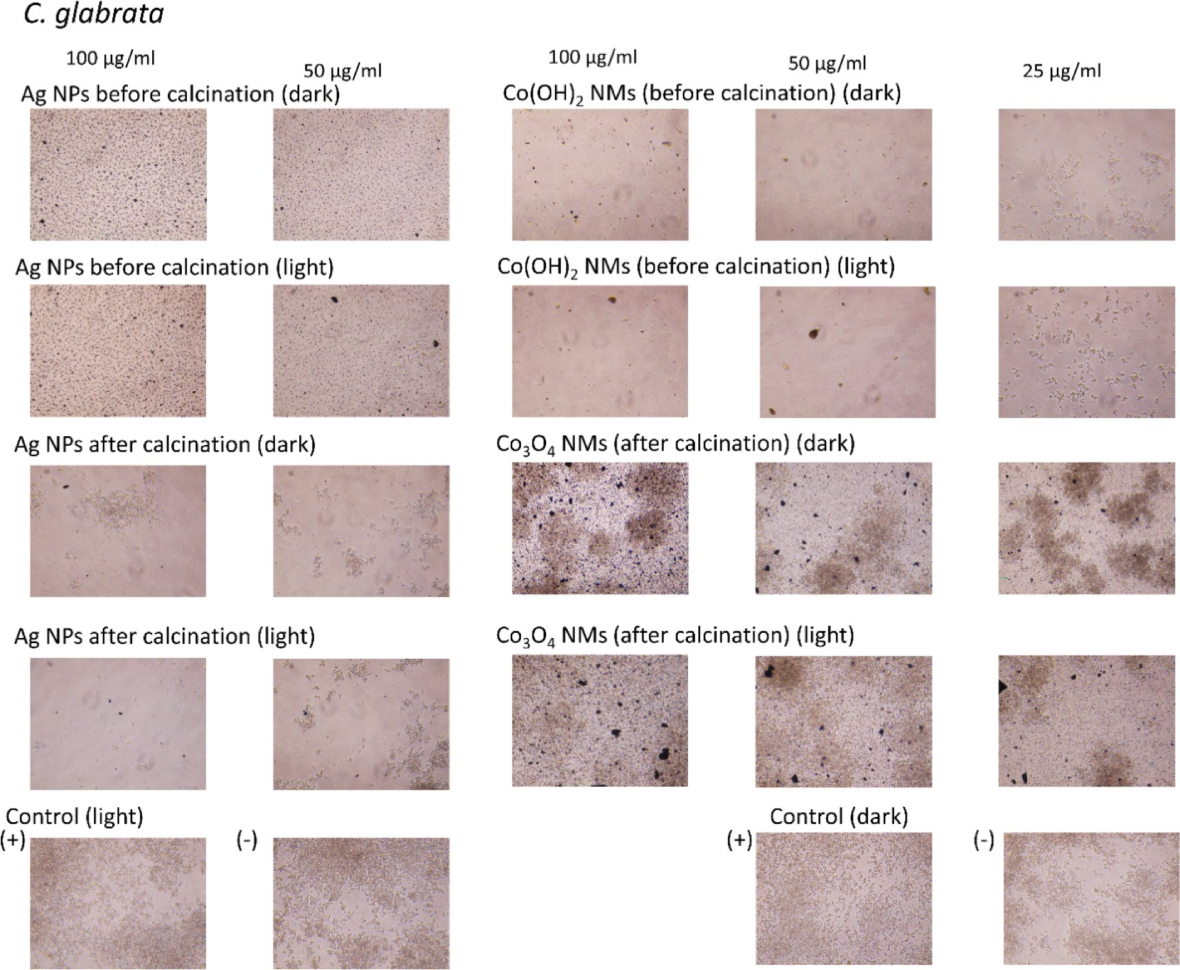

Supplement: S5 Fig — (TIFF) [file pone.0274753.s005.tiff]

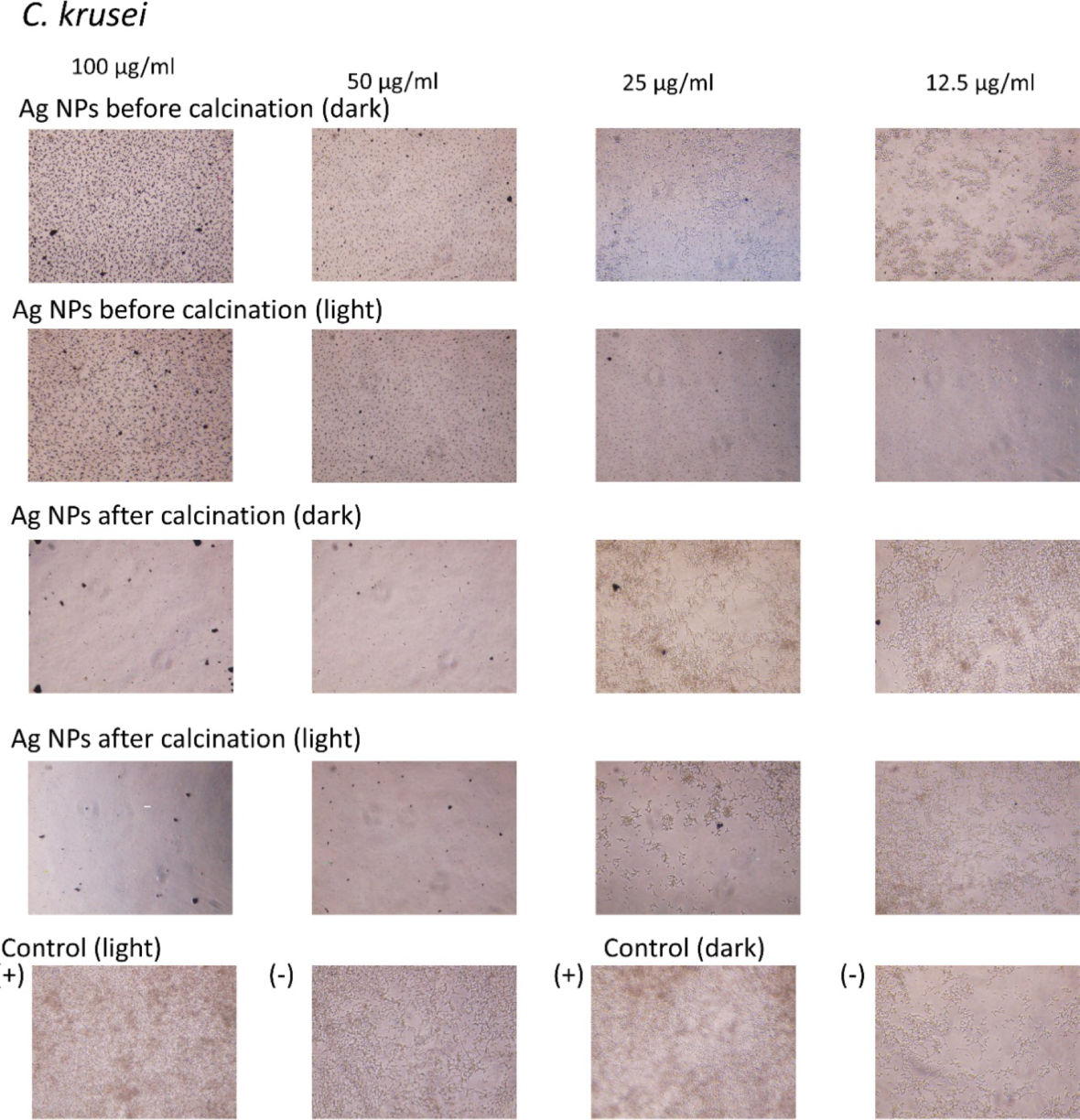

Supplement: S6 Fig — (TIFF) [file pone.0274753.s006.tiff]
