# Supplementary material for: Characterization of nanomaterials synthesized from Spirulina platensis extract and their potential antifungal activity
Source: PLoS One. 2022 Sep 16;17(9):e0274753. doi: 10.1371/journal.pone.0274753 (PMC9481030; doi:10.1371/journal.pone.0274753)
Supplement: S2 Table — (DOCX) [file pone.0274753.s008.docx]

| **BEFORE SYNTHESIS** | | | **MIMICKING** | | | **AFTER SYNTHESIS** | | |
| --- | --- | --- | --- | --- | --- | --- | --- | --- |
| **Peak Name** | **RT (min)** | **Relative Area (%)** | **Peak Name** | **RT (min)** | **Relative Area (%)** | **Peak Name** | **RT (min)** | **Relative Area (%)** |
| 1,4-Bis(trimethylsilyl)benzene | 6.105 | 0.03 | 1,4-Bis(trimethylsilyl)benzene | 6.128 | 0.04 | 1,4-Bis(trimethylsilyl)benzene | 6.077 | 1.98 |
| Ethylbenzene | 6.692 | 0.00 | Methylamine, | 7.676 | 0.13 | Methylphosphonic acid | 6.450 | 0.75 |
| Methylamine | 7.706 | 0.10 | 2-Methylpentan-2-ol, | 8.183 | 0.02 | Ethylbenzene | 6.637 | 11.90 |
| 2-Methylpentan-2-ol | 8.187 | 0.02 | Nonane | 8.690 | 0.02 | 1,4-Dimethylbenzene | 7.011 | 24.99 |
| Nonane | 8.694 | 0.02 | Diethylamine | 11.519 | 0.03 | 2-Methylpentan-2-ol | 8.105 | 1.32 |
| Ethyl mandelate | 9.201 | 0.02 | Ethanamine | 11.733 | 0.10 | Nonane | 8.638 | 1.21 |
| Diethanolamine, | 11.496 | 0.04 | Ethylene glycol | 13.361 | 0.03 | Ethyl mandelate | 9.201 | 0.02 |
| Ethanamine, | 11.709 | 0.08 | Lactic Acid | 15.469 | 0.04 | Ethylene glycol | 13.335 | 9.41 |
| 4-Methylvaleric acid | 12.030 | 0.01 | Glycolic acid | 17.123 | 0.18 | Propylene glycol | 13.869 | 0.99 |
| Ethylene glycol, | 13.337 | 0.27 | Glycerol, | 19.418 | 0.30 | Glycerol, | 19.420 | 1.81 |
| Propylene glycol | 13.871 | 0.04 | Glycine | 19.845 | 0.05 | Glyceryl-glycoside | 30.895 | 1.01 |
| Benzenemethanol, α-[(methylamino)methyl]- | 14.218 | 0.01 | Glyceryl-glycoside | 30.894 | 0.59 | 1-Monopalmitin | 32.844 | 1.04 |
| 3-Pyridinol, | 14.672 | 0.01 | 1-Monopalmitin | 32.842 | 0.05 |  |  |  |
| Lactic Acid | 15.472 | 0.01 | Lactose | 34.336 | 0.11 |  |  |  |
| Glycerol | 19.422 | 0.07 |  |  |  |  |  |  |
| Butanedioic acid | 19.929 | 0.03 |  |  |  |  |  |  |
| 6-Dimethyl(trimethylsily)silyloxytetradecane | 21.210 | 0.01 |  |  |  |  |  |  |
| L-Glutamic acid, bis(trimethylsilyl) ester | 22.864 | 0.07 |  |  |  |  |  |  |
| Glycerol phosphate | 29.830 | 0.04 |  |  |  |  |  |  |
| Glyceryl-glycoside TMS ether | 30.417 | 0.05 |  |  |  |  |  |  |
| Glyceryl-glycoside TMS ether | 30.897 | 0.61 |  |  |  |  |  |  |
| Lactose, | 34.340 | 0.06 |  |  |  |  |  |  |
| B-D-Lactose | 37.382 | 0.05 |  |  |  |  |  |  |
