# Supplementary material for: Characterization of nanomaterials synthesized from Spirulina platensis extract and their potential antifungal activity
Source: PLoS One. 2022 Sep 16;17(9):e0274753. doi: 10.1371/journal.pone.0274753 (PMC9481030; doi:10.1371/journal.pone.0274753)
Supplement: S3 Table — (DOCX) [file pone.0274753.s009.docx]

| **BEFORE SYNTHESIS** | | | **MIMICKING** | | | **AFTER SYNTHESIS** | | |
| --- | --- | --- | --- | --- | --- | --- | --- | --- |
| **Peak Name** | **RT (min)** | **Relative Area (%)** | **Peak Name** | **RT (min)** | **Relative Area (%)** | **Peak Name** | **RT (min)** | **Relative Area (%)** |
| 1,4-Bis(trimethylsilyl)benzene | 6.105 | 0.03 | 1,4-Bis(trimethylsilyl)benzene | 6.075 | 0.03 | Methylphosphonic acid | 6.235 | 0.00 |
| Ethylbenzene | 6.692 | 0.00 | Methylamine, | 7.703 | 0.09 | 2-Methylpentan-2-ol | 7.970 | 0.03 |
| Methylamine | 7.706 | 0.10 | 2-Methylpentan-2-ol | 8.156 | 0.02 | Ethyl mandelate | 8.904 | 0.01 |
| 2-Methylpentan-2-ol | 8.187 | 0.02 | Nonane | 8.690 | 0.02 | 1-Pentamethyldisilyloxytridecane | 9.037 | 0.01 |
| Nonane | 8.694 | 0.02 | Ethyl mandelate | 9.201 | 0.02 | B-D-Glucopyranoside, methyl 2-(acetylamino)-2-deoxy-3-O-(trimethylsilyl)-, cyclic methylboronate | 9.758 | 0.00 |
| Ethyl mandelate | 9.201 | 0.02 | Diethylamine | 11.492 | 0.02 | Cystathionine | 10.372 | 0.00 |
| Diethanolamine | 11.496 | 0.04 | Ethanamine | 11.706 | 0.09 | Ethanamine | 11.493 | 0.01 |
| Ethanamine | 11.709 | 0.08 | Ethylene glycol | 13.334 | 0.14 | N,N-Diethyl(trimethylsilyl)carbamate | 15.175 | 0.02 |
| 4-Methylvaleric acid | 12.030 | 0.01 | Propylene glycol | 13.867 | 0.02 | Propylamine | 16.857 | 0.02 |
| Ethylene glycol | 13.337 | 0.27 | Lactic Acid | 15.469 | 0.03 | Mercaptoethanol | 17.310 | 0.00 |
| Propylene glycol | 13.871 | 0.04 | Glycolic acid | 15.789 | 0.01 | Butanedioic acid | 19.872 | 0.00 |
| Benzenemethanolα-[(methylamino)  methyl] | 14.218 | 0.01 | Urea | 17.123 | 0.12 | Retinal | 21.714 | 0.00 |
| 3-Pyridinol | 14.672 | 0.01 | Glycerol | 19.418 | 0.35 | Palmitic acid | 28.172 | 0.00 |
| Lactic Acid | 15.472 | 0.01 | Butanedioic acid | 19.952 | 0.05 |  |  |  |
| Glycerol | 19.422 | 0.07 | 6-Dimethyl(trimethylsily)silyloxytetradecane | 21.210 | 0.01 |  |  |  |
| Butanedioic acid | 19.929 | 0.03 | L-Glutamic acid, bis(trimethylsilyl) ester | 22.861 | 0.05 |  |  |  |
| 6-Dimethyl(trimethylsily)silyloxytetradecane | 21.210 | 0.01 | β-D-Xylopyranose | 28.065 | 0.04 |  |  |  |
| L-Glutamic acid, bis(trimethylsilyl) ester | 22.864 | 0.07 | Myo-Inositol | 28.946 | 0.02 |  |  |  |
| Glycerol phosphate | 29.830 | 0.04 | 5,8,11-Eicosatrienoic acid, (Z)-, TMS derivative | 29.586 | 0.07 |  |  |  |
| Glyceryl-glycoside TMS ether | 30.417 | 0.05 | 9,12-Octadecadienoic acid (Z,Z) | 29.746 | 0.18 |  |  |  |
| Glyceryl-glycoside TMS ether | 30.897 | 0.61 | Glyceryl-glycoside | 30.413 | 0.09 |  |  |  |
| Lactose, | 34.340 | 0.06 | Glyceryl-glycoside | 30.894 | 0.84 |  |  |  |
| B-D-Lactose | 37.382 | 0.05 | Lactose | 34.336 | 0.09 |  |  |  |
