# Supplementary material for: Characterization of nanomaterials synthesized from Spirulina platensis extract and their potential antifungal activity
Source: PLoS One. 2022 Sep 16;17(9):e0274753. doi: 10.1371/journal.pone.0274753 (PMC9481030; doi:10.1371/journal.pone.0274753)
Supplement: Attachment — Submitted filename: Reviewing the manuscript (PONE-D-22-13061).docx [file pone.0274753.s010.docx]

The manuscript introduces the details on the synthesis and full characterization of a number of nanomaterial structures using a green chemistry approach, with evaluating the activity of candidate particles against three *Candida* species. Some suggestions are listed below for enhancing the presentation of the work:

**Abstract**
-It is better to involve one or two sentences on the choice of the particular tested nanoparticles.

-The last sentence: should be splitted into two or more sentences with giving the exact values of MIC and the meaning of this abbreviation.

**Introduction**
-Very long section, specially the Ag NPs part, and should be summarized with transferring of a part to the discussion section.

**Methodology**
-Paragraph starting with (It should be noted that the trace …..) should be moved to the first paragraph under this section after the sentence beginning with (The medium was prepared according….).
**-Synthesis of nanostructures:** How much metal salts were added to the extract to synthesize each type of particles? Were they added as powder or in solution?
**-Materials characterization:** The sentence beginning with (Then, a 1.25 M HCl solution was ….) is not clear. Was the initial pH in case of titanium NPs zero?

**Results**
-Generally, the units are inconsistent between tables and the main text. No statistical analysis was performed, particularly for the experiments assessing the inhibitory concentrations of nanomaterials.
**-Fungal study & Table 4:** There are no details about any control groups studied. Is there any effect of light only on the tested fungi?
-The supplementary figures & tables are not present in the uploaded version.
